# Supplementary material for: Associations between podoconiosis and pedogenic factors globally – A systematic review
Source: PLoS Negl Trop Dis. 2025 Jul 7;19(7):e0013294. doi: 10.1371/journal.pntd.0013294 (PMC12270302; doi:10.1371/journal.pntd.0013294)
Supplement: S3 File — (DOCX) [file pntd.0013294.s003.docx]

**AXIS assessment**

| **Study** | **Year** | **Total score quality** **(0-20)** |
| --- | --- | --- |
| ***Cooper and Nick*** | 2023 | 19 |
| ***Cooper et al.*** | 2019 | 19 |
| ***Corachan et al.*** | 1988 | 8 |
| ***Crivelli*** | 1986 | 13 |
| ***Deribe et al.*** | 2015 | 20 |
| ***Deribe et al.*** | 2018 | 19 |
| ***Deribe et al.*** | 2023 | 19 |
| ***de Lalla et al.*** | 1978 | 7 |
| ***Frommel et al.*** | 1993 | 14 |
| ***Gislam et al.*** | 2020 | 20 |
| ***Kebede*** | 2009 | 13 |
| ***Kihembo et al.*** | 2017 | 17 |
| ***Lalla et al.*** | 1988 | 14 |
| ***Molla et al.*** | 2014 | 19 |
| ***Muli et al.*** | 2017 | 18 |
| ***Negasa and Dufera*** | 2021 | 18 |
| ***Onapa et al.*** | 2001 | 16 |
| ***Price*** | 1974 | 12 |
| ***Price*** | 1976a | 14 |
| ***Price*** | 1976b | 14 |
| ***Price and Bailey*** | 1984 | 15 |
| ***Price and Heather*** | 1972 | 7 |
| ***Price and Henderson*** | 1978 | 7 |
| ***Price and Henderson*** | 1979 | 7 |
| ***Price and Henderson*** | 1980 | 10 |
| ***Price et al.*** | 1981 | 7 |
| ***Spooner and Davies*** | 1981 | 10 |
